# Provision of Hospital Accommodation

**Published:** 1921-01-29

**Authors:** 


### PROVISION OF HOSPITAL ACCOMMODATION.

By AN INFIRMARY MEDICAL SUPERINTENDENT.

### I.—THE PROBLEM.

DISEASE and injury are two of the means which Nature uses to rid the world of those who are not suited to their surroundings. If we were content to give Nature free scope, if we could keep our muddling fingers from trying to improve upon her ways, if we would let her misfits die or recover by their unaided efforts, then Nature would no doubt in time bring forth a race, so wary that no accident could befall them, so tissued that no disease could find an entry to their bodies. Such beings would live until the energy with which they started fell below the level needful for life, and then death would clear them out to make room for Nature's fresh experiments.

Why man ever set himself to try to better Nature's ways is a problem to be left for the materialist to solve if he can. He may point to its beginnings in the need to cherish the young life and to protect the mother during pregnancy and suckling, to its extension to infectious disease in order to safeguard the uninfected, and to its final developments with the realisation of Society as a machine dependent for its well-being upon the soundness of each of its parts. Even so, it would have been simple, more in accord with Nature's way, more economical and less troublesome if, instead, man had decided to kill the diseased and maimed so soon as they became useless.

> I heard a devil curse
> Over the heath and the furse :
> "Mercy could be no more
> If there were nobody poor,
> And pity no more could be
> If all were happy as ye."

That was Blake's third and last pronouncement: perhaps if he had written a fourth, he would have put the words in the mouth of the Angel.

### LIMITATIONS OF THE STATE HOSPITAL.

But, for good or for evil, man's conscience has chosen the more difficult way. When the idea of beneficent and malevolent powers ruling man's destinies arose, it was natural to look upon health and disease as rewards and punishments given by the gods. It was natural, too, that the sick should come to the temples to be healed by the gods through the mediation of the priests. So the hospital came into being. Financed at first by the gifts of the sick themselves, it soon became necessary to find other sources of revenue, and gifts from the devout supplied them. Later arose hospitals, detached from the churches, served by men trained in medical work and supported by the charitable. Lastly, the State took a hand.

Realising that in an organised system of society the poor had claims upon the more wealthy, that the control of infection was necessary to prevent epidemics, that voluntary efforts could deal with only a part of the sick, the State set up hospitals paid for out of the rates. The difference in the financing of the two classes of hospitals led to an important difference in their methods. The managers of the voluntary hospitals felt they were justified in extracting all the money they could from the charitable and in spending it freely for the relief of the sick who came to them; the elected representatives of the taxpayer, dealing with money raised by taxation, felt they were in duty bound to cut their expenditure down to the lowest limit possible without outraging the hardly awakened social conscience. So, in Britain, at any rate, the rate-supported hospital has always lagged behind the voluntary, in staffing, in equipment, and in research work.

### A CHANGED OUTLOOK.

Now both kinds of hospitals are faced by new difficulties and by new problems. The voluntary hospitals can no longer raise the money they need; they can no longer supply the beds required by those whose wants they were intended to meet. The rate-supported hospitals can no longer pretend before an educated public to deal efficiently with their sick with the meagre equipment and staff they have hitherto used. So voluntary hospitals are closing or are threatening to close some of their wards; and the rate-supported hospitals throughout the country are at last being equipped for the simpler special methods of diagnosis and treatment, are increasing their staffs and are beginning to add to their staffs a few—in most cases a very few—specialists. And, with these changes, there has arisen a demand for hospital accommodation from people who can find no room in the voluntary hospitals and who will not enter the ordinary rate-supported hospital because of its association with the Poor Law.

The solution of the problem of providing efficient hospitals for those who need them is greatly complicated by the haphazard way in which our present hospital system has arisen. It is not helped by those who, in the reforms they advocate, seek to grind their own axes or to aggrandise that part of the system in which they work gratuitously or for payment. Many of the writers and speakers on the subject seem to be possessed with the idea of maintaining the supply of charitable contributions and the voluntary hospital as now organised, or of keeping in existence Boards of Guardians, or of providing for the medical education of the student, the practitioner, and the budding specialist, or of increasing the material for medical research—the cure of the sick coming in merely as a means to one or more of these ends.

### THE LACK OF CO-ORDINATION.

At present the public provision for the detection, prevention, and treatment of disease is in the hands of a very large number of different and

**Provision of Hospital Accommodation**—(*continued*)

unconnected bodies, but the provision of hospitals is mainly in the hands of three sets of people—the charitable public, who provide the voluntary hospitals; the Boards of Guardians, who provide the Poor-law infirmaries; and the County Councils, who provide special hospitals chiefly for the infectious fevers, for mental diseases, and in some cases for consumption. These three bodies are all acting independently of one another, with practically no interchange of information and with no one to correlate their activities. More than this, the hospitals of any one class work, as a rule, independently of the other hospitals in the same class. If the Poor-law infirmary in one parish is temporarily overcrowded it cannot send its surplus patients to an infirmary in an adjacent parish, unless an agreement has been made between the two Boards of Guardians. Similarly, if an infirmary has equipment and staff for specialised forms of treatment it cannot receive suitable patients from adjacent parishes. The lack of any system of interchange of information between the various Boards of Guardians leads to one Board knowing very little about the provision made by other Boards. It is obvious that this lack of co-ordination prevents the full use to the best advantage of the hospital accommodation that exists.

The Hospitals' Only Difficulty.

Before trying to devise a scheme to remedy the admitted shortage of hospital beds, it is necessary, first, to inquire whether the existing hospitals are worked in such a way as to ensure that their beds are used to the greatest advantage. Most of the voluntary hospitals have reached in their treatment of indoor patients a very high degree of efficiency. They are well staffed; they are well equipped; and their patients are discharged as soon as convalescent. They escape many difficulties because they deal only with patients acutely ill; they transfer to other institutions the chronic patient; and they discharge a patient who is able to be up and about, even if he is not sufficiently well to return to his usual work. Their only difficulty is want of funds—a want which has led to the closing of a number of their wards. Given the money, these wards could be reopened and would provide us with a very useful addition to our resources, but the number of beds so obtained would not go far towards meeting the demand. The hospitals of the County Councils may for the moment be ignored. Dealing as they do with special types of diseases, their use is limited to the diseases in which they specialise and their beds cannot be utilised for the treatment of other patients, unless we radically alter our methods of dealing with the insane and with infectious patients. It is to the Poor-law infirmary we must turn in the hope of finding the beds we want. And the first thing to determine is, whether the beds in these infirmaries are fully used, and are used with the greatest possible efficiency.

This we propose to consider in the two following articles.

